# Influence of perioperative SARS-CoV-2 infection on mortality in orthopaedic inpatients with surgically treated traumatic fractures

**DOI:** 10.1007/s00590-022-03226-x

**Published:** 2022-03-24

**Authors:** Mathias Granqvist, Pontus Hedberg, Pontus Nauclér, Anders Enocson

**Affiliations:** 1 Department of Trauma, Acute Surgery and Orthopaedics, Karolinska University Hospital, Stockholm, Sweden; 2 Department of Infectious Diseases, Karolinska University Hospital, Stockholm, Sweden; 3 Department of Molecular Medicine and Surgery, Karolinska Institutet, Stockholm, Sweden; 4 Department of Medicine, Division of Infectious Diseases, Karolinska Institutet, Solna, Stockholm, Sweden

**Keywords:** Orthopaedic surgery, Fracture, SARS-CoV-2, COVID-19, 30-day mortality

## Abstract

**Background:**

SARS-CoV-2 has had an extensive influence on orthopaedic surgery practice and has been associated with an increased risk of mortality. There is limited evidence of how this pertains to acute orthopaedic surgery with inpatient care.

**Methods:**

A retrospective cohort study on traumatic fracture patients requiring inpatient care between February 25, 2020 and March 25, 2021 was conducted. Patients were grouped by perioperative SARS-CoV-2 infection, defined as a positive SARS-CoV-2 test from 7 days before to 7 days after orthopaedic surgery, and compared using linear regression and Cox proportional hazards model for primary outcome 30-day mortality and secondary outcome hospital length of stay.

**Results:**

In total, 5174 adults with a length of stay ≥ 48 h and an orthopaedic procedure due to a registered traumatic fracture were admitted from February 25, 2020 and discharged before March 26, 2021. Among the 5174 patients, 65% (3340/5174) were female, 22% (1146/5174) were 60–74 years and 56% (2897/5174) were 75 years or older. In total, 144 (3%) had a perioperative SARS-CoV-2 infection. Perioperative SARS-CoV-2 infection was associated with an increased 30-day mortality (aOR 4.19 [95% CI 2.67–6.43], *p* < 0.001). The median (IQR) length of stay after surgery was 13 days (IQR 6–21) for patients with, and 7 days (IQR 2–13) for patients without, perioperative SARS-CoV-2 infection.

**Conclusions:**

Perioperative SARS-CoV-2 infection increased 30-day mortality risk and hospital length of stay for traumatic fracture patients requiring inpatient surgical care. Pre- and postoperative infection were both associated with similar increases in mortality risk.

**Supplementary Information:**

The online version contains supplementary material available at 10.1007/s00590-022-03226-x.

## Introduction

The pandemic dissemination of severe acute respiratory syndrome coronavirus 2 (SARS-CoV-2) has had an extensive influence on orthopaedic surgery practice in affected countries. Since the first reported cases of SARS-CoV-2 in December 2019, relative rates of orthopaedic surgeries have changed, with a general decrease in traumatic fractures across reporting countries [1–4]. The relative frequencies of fractures have also changed, with lower rates of fractures resulting from high energy trauma and unchanged or slightly higher rates of fractures related to fragility [5].

Mortality rates in patients infected with SARS-CoV-2 have been estimated to 0.6% increasing to 4.3% in patients over 70 years of age [6]. This increased risk of mortality associated with SARS-CoV-2 infection seems to also pertain to patients presenting with orthopaedic traumatic injuries. Postoperative mortality rate nearly doubled among patients undergoing orthopaedic surgery with either pre- or postoperative SARS-CoV-2 infection in the IMPACT-Restart study [7]. Initially, COVID-19 negative patients requiring urgent orthopaedic surgery that turned COVID-19 positive within 30 days of surgery had 30% higher 30-day mortality [8].

Within specific diagnoses of orthopaedic surgery such as hip fractures, findings are consistent. In a study by Hall et al., COVID-19 positivity in conjunction with hip fracture was associated with 65% increased 30-day mortality [9]. Findings from a prospective single-centre study were similar, showing a 2.4-fold increase in mortality [10]. Treatment of hip fractures in hospitals with SARS-CoV-2 patients has not had an impact on mortality rates in general, however, when comparing to prior years [11]. The influence of perioperative SARS-CoV-2 infection patients requiring inpatient fracture surgery on postoperative mortality especially across European countries remains scarce.

The aims of this study were to assess the impact of SARS-CoV-2 on 30-day mortality and hospital length of stay in traumatic fracture patients presenting with positive SARS-CoV-2 samples across all hospitals in Stockholm County.

## Methods

### Study population

We conducted a population-based observational cohort study of all adult inhabitants in Stockholm County, Sweden, undergoing an inpatient orthopaedic procedure in Stockholm County between February 25, 2020 and March 25, 2021. Inclusion criteria were age ≥ 18 years an inpatient orthopaedic procedure performed due to a traumatic fracture. Only the first included health care episode was considered for each patient. Hospitalizations with the patient being discharged alive within 48 h were excluded. The study was approved by the Swedish Ethical Review Board (Dnr 2018/1030-31, COVID-19 research amendment Dnr 2020-01385).

### Data collection and definitions

Data on diagnoses, medical procedures, outpatient and inpatient contacts and demographics were obtained from a health data register for the population of the Stockholm County (VAL; Stockholm regional healthcare data warehouse) that contains administrative data from in- and outpatient care in Stockholm County. Data were linked to SARS-CoV-2 PCR positive results, extracted from the Public Health Agency of Sweden database—SmiNet, where it is mandatory to report all SARS-CoV-2 positive tests, using unique personal identification numbers.

Patient selection was based on nationally harmonized procedure code registered in the medical records according to KVA, which is a Swedish classification system of care measures. Included KVA classifications are further specified in Table S1. Patients were grouped by perioperative SARS-CoV-2 infection positivity, defined as occurrence of positive SARS-CoV-2 PCR-tests 7 days before to 7 days after an orthopaedic procedure. Specific co-morbidities were assessed from 5 years up to 1 day before the included hospitalization (Table S2). The COVID-19 pandemic was divided into the first wave (before August 1, 2020), between first and second wave (August 1, 2020 to September 30, 2020), second wave (October 1, 2020 to January 31, 2021) and after second wave (February 1, 2021 to March 25, 2021).

### Data collection and outcomes

Outcomes were specified before data collection and analysis. The primary outcome was 30-day mortality after orthopaedic procedure, with the day of the orthopaedic procedure defined as day 0. Secondary outcome was length of stay after orthopaedic procedure.

### Statistical analyses

The study was done in accordance with STROBE guidelines for observational studies [12]. Patients were grouped according to presence of perioperative SARS-CoV-2 infection or not. Continuous data were compared between groups using Wilcoxon rank sum test (Mann–Whitney *U*) and for categorical data Chi-square or Fisher’s Exact test depending on sample size.

All outcome measures were analysed using univariate and multivariate regression analyses. One multivariate regression model was defined a priori, adjusting for age (≤ 65 years vs ≥ 65 years), sex, procedure type, hospital where the first orthopaedic procedure was performed and number of co-morbidities. Mortality was analysed using logistic regression with odds ratios (OR) and 95% confidence interval (CI) presented, whereas length of stay was analysed using Fine and Gray models for hospital discharge alive (event of interest) and death (competing risk) with subdistribution hazard ratios (SHR) and 95% CI presented. The Kaplan–Meier estimator was used to visualize the probability of survival and the Aalen-Johansen estimator for length of stay with death as competing event.

In order to address potential important confounding, the following predefined sensitivity analyses were performed; restriction to (1) hospital admissions during the first wave of the COVID-19 pandemic and (2) hospital admissions during the second wave of the COVID-19 pandemic. One post hoc analysis was added; restriction to (4) patients with hip, femur or pelvic surgery performed.

All statistical analyses and data processing were performed in R, version 4.0.3 (R Foundation).

## Results

**Fig. 1:**
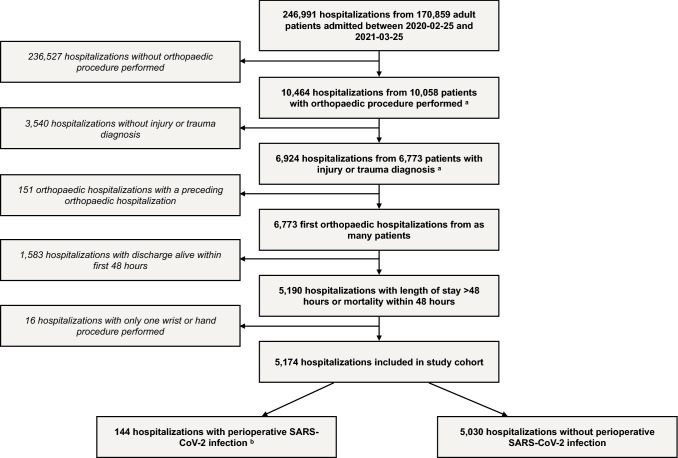
Study flow chart. Flow chart of hospitalizations with orthopaedic procedures performed. ^a^See Table S1 for included orthopaedic procedures and diagnoses. ^b^Defined as occurrence of positive SARS-CoV-2 PCR-tests 7 days before to 7 days after an orthopaedic procedure

In total, 246,991 hospitalizations in 170,859 adult patients were admitted from February 25, 2020 and discharged before March 26, 2021. The final study cohort consisted of 5174 admissions from as many patients with a length of stay ≥ 48 h and an orthopaedic procedure with injury or trauma diagnosis registered (Fig. 1). Among the 5174 patients, 65% (3340/5174) were female, 22% (1146/5174) were 60–74 years and 56% (2897/5174) were 75 years or older.

### Overview of the occurrence of perioperative SARS-CoV-2 infection

Three per cent (144/5174) of the patients had perioperative SARS-CoV-2 infection (a positive SARS-CoV-2 test from 7 days before up to 7 days after orthopaedic procedure). Among these 144 patients, 56% (81/144) had a positive test up to the procedure date and 44% (63/144) 1–7 days after the procedure. The proportion of orthopaedic procedures with perioperative SARS-CoV-2 infection clearly followed the distribution of the first and second COVID-19 wave in Stockholm, with April, 2020 having the highest proportion of perioperative SARS-CoV-2 infection (7%, 25/346) (Fig. S1).

### Baseline characteristics and orthopaedic procedures

Patients with perioperative SARS-CoV-2 infection were, compared to patients without perioperative SARS-CoV-2 infection, older, more often immunocompromised and more often had chronic kidney disease, chronic heart disease and neurological disease (Table 1). Regarding orthopaedic procedures performed, patients with perioperative SARS-CoV-2 infection more often had pelvis-, hip- or femur-related surgery performed, and the proportion undergoing multiple procedures was higher compared to patients without perioperative SARS-CoV-2 infection.

**Table 1:** Baseline characteristics and orthopaedic procedures in patients with and without perioperative SARS-CoV-2 infection

Characteristic	Perioperative SARS-CoV-2		*P* Value
	Yes (*n* = 144)	No (*n* = 5030)	
Female sex, *n* (%)	86 (60)	3254 (65)	0.254
Age, median (IQR)	83 (74–89)	77 (62–86)	< 0.001
Age category, *n* (%)			
18–29	4 (3)	189 (4)	< 0.001
30–44	6 (4)	331 (7)	
45–59	9 (6)	592 (12)	
60–74	21 (15)	1125 (22)	
≥75	104 (72)	2793 (56)	
Nursing home residency, *n* (%)	33 (23)	633 (13)	< 0.001
Previous co-morbidities, *n* (%)			
Cancer	26 (18)	833 (17)	0.816
Cerebrovascular disease	19 (13)	563 (11)	0.538
Chronic kidney disease	27 (19)	489 (10)	0.001
Diabetes mellitus (type 1 or 2)	26 (18)	736 (15)	0.306
Heart disease	63 (44)	1,534 (31)	0.001
Immunosuppression	53 (37)	1,385 (28)	0.019
Liver disease	7 (5)	123 (2)	0.120
Lung disease	29 (20)	981 (19)	0.934
Neurological disease	70 (49)	1,888 (38)	0.009
Obesity	7 (5)	207 (4)	0.817
Substance use disorder	11 (8)	473 (9)	0.567
No. co-morbidities, median (IQR)	2 (1–3)	2 (0–3)	< 0.001
No. co-morbidities, *n* (%)			
None	24 (17)	1280 (25)	< 0.001
1–2	61 (42)	2206 (44)	
≥ 3	59 (41)	1544 (31)	
COVID-19 wave, *n* (%)			
First wave	66 (46)	1923 (38)	< 0.001
Between first and second wave	5 (4)	759 (15)	
Second wave	63 (44)	1648 (33)	
After second wave	10 (7)	700 (14)	
Length of stay before orthopaedic procedure, median (IQR)	1 (1–2)	1 (0–2)	0.038
Procedure type, *n* (%)			
Elbow, forearm	1 (1)	200 (4)	0.002
Foot, ankle	15 (10)	819 (16)	
Hip, femur, pelvis	106 (74)	2906 (58)	
Knee, lower leg	4 (3)	392 (8)	
Shoulder, arm	6 (4)	354 (7)	
Spinal	0 (0)	35 (1)	
Wrist, hand	2 (1)	101 (2)	
Multiple procedure types	10 (7)	223 (4)	

### Clinical outcomes in patients with and without perioperative SARS-CoV-2 infection

**Fig. 2:**
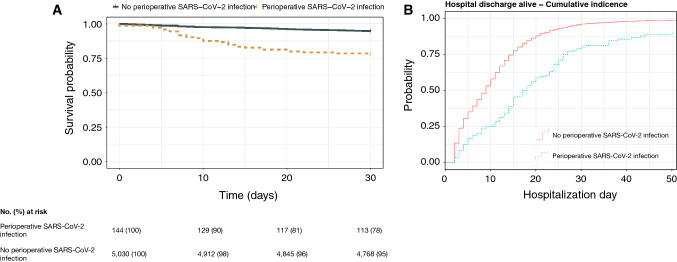
Mortality and length of stay after surgery in patients with and without perioperative SARS-CoV-2 infection. **a** Unadjusted Kaplan–Meier curves and risk tables for 30-day mortality in patients with and without perioperative SARS-CoV-2 infection. **b** Cumuative risk (incidence) of hospital discharge alive in patients with and without perioperative SARS-CoV-2 infection

The 30-day mortality after orthopaedic procedure was 22% (32/144) in patients with perioperative SARS-CoV-2 infection, compared to 5% (267/5,030) in patients without perioperative infection (Table 2). The median time to mortality was 13 days (IQR 6–21) for patients with perioperative SARS-CoV-2 infection, compared to 10 days (IQR 7–14) for patients without perioperative SARS-CoV-2 infection (Fig. 2a). Perioperative SARS-CoV-2 infection was associated with an increased 30-day mortality (OR 5.10 [95% CI 3.33–7.61], *p* < 0.001), also when adjusting for age, sex, procedure type, hospital, phase of the COVID-19 pandemic and number of co-morbidities (aOR 4.19 [95% CI 2.67–6.43], *p* < 0.001). The association with an increased 30-day mortality remained when restricting the analysis to patients admitted during the first wave (aOR 5.69 [95% CI 2.94–10.66], *p* < 0.001) and second wave (aOR 3.78 [95% CI 1.87–7.31], *p* < 0.001) of the pandemic, as well as when restricting the analysis to patients with hip, femur or pelvic surgery performed (aOR 4.07 [95% CI 2.53–6.41], *p* < 0.001).

**Table 2:** Mortality and length of stay after orthopaedic procedures in patients with and without perioperative SARS-CoV-2 infection

Outcome variable	Perioperative SARS-CoV-2		Unadjusted ratio (95% CI) ^a^	Adjusted ratio (95% CI)^a,b^
	Yes	No		
Main analysis, cohort size *n*	144	5,030		
Mortality, 30-day, *n* (%)	32 (22)	267 (5)	5.10 (3.33–7.61)	4.19 (2.67–6.43)
Length of stay after surgery, median (IQR)	13 (6–21)	7 (2–13)	0.47 (0.40–0.55)	0.49 (0.41–0.59)
First wave of the COVID-19 pandemic, cohort size *n*	66	1923		
Mortality, 30-day, *n* (%)	17 (26)	107 (6)	5.89 (3.20–10.38)	5.69 (2.94–10.66)
Length of stay after surgery, median (IQR)	13 (7–21)	7 (2–13)	0.46 (0.37–0.56)	0.45 (0.34–0.60)
Second wave of the COVID-19 pandemic, cohort size *n*	63	1648		
Mortality, 30-day, *n* (%)	14 (22)	90 (6)	4.95 (2.55–9.07)	3.78 (1.87–7.31)
Length of stay after surgery, median (IQR)	13 (7–22)	7 (2–13)	0.45 (0.35–0.57)	0.52 (0.41–0.67)
Hip, femur or pelvic surgery, cohort size n	106	2906		
Mortality, 30-day, *n* (%)	29 (27)	243 (8)	4.13 (2.60–6.38)	4.07 (2.53–6.41)
Length of stay after surgery, median (IQR)	13 (6–20)	10 (5–15)	0.50 (0.40–0.62)	0.50 (0.39–0.63)

^a^Mortality was analysed by logistic regression, length of stay was analysed by Fine and Gray models

^b^Adjusted for age (< 65 years vs ≥ 65 years), sex, procedure type, hospital where first procedure was performed and no. co-morbidities. For the sensitivity analysis including only hip, femur or pelvic surgery, procedure type was excluded from the adjusted model

The median (IQR) length of stay after orthopaedic procedure was 13 days (IQR 6–21) and 7 days (IQR 2–13) in patients with and without perioperative SARS-CoV-2 infection, respectively (Fig. 2b). The subdistribution hazard ratio for hospital discharge alive was lower in patients with perioperative SARS-CoV-2 infection, compared to patients without perioperative SARS-CoV-2 infection (aSHR 0.49 [95% CI 0.41–0.59], *p* < 0.001). This was consistent across all sensitivity analyses performed.

## Discussion

We here compared outcomes for traumatic fracture patients based on SARS-CoV-2 positivity in a population-based, retrospective observational cohort study and found a significantly higher mortality risk and increased hospital length of stay that remained after adjusting for covariates. These findings are in accordance with prior studies also on magnitude of effect which indicates that the influence of infection on fracture patients is consistent across countries [7, 9]. The increased mortality rate also remained across both “waves” of increased SARS-CoV-2 rates in Stockholm and for all specified subsets of fracture groups. Collectively, the findings on mortality rate have important implications for the perioperative treatment of fracture patients.

With a median age of 83 and 77 for SARS-CoV-2-positive and -negative patients, respectively, based on prior findings, these patients are at a high risk of mortality and morbidity from infection [13]. Comparing the 30-day mortality for SARS-CoV-2-positive fracture patients to inpatients > 80 years in other population-based cohorts reveals a still-higher mortality risk in this cohort with acute fractures concomitantly ([14]; 4.19 (crude 5.10) vs 3.17). This suggests an additive mortality risk for these patients that needs further inquiry such as cause of death to possibly mitigate.

As SARS-CoV-2 infection occurred in 44% of patients postoperatively and still conferred an increased mortality risk, this highlights the need for stringent avoidance of infection spreading at the wards. General clinical guidelines to decrease SARS-CoV-2 infection rates for patients and health care workers have been proposed [15]. Given the magnitude of risk increase for mortality, SARS-CoV-2 status should be taken into consideration also in patients with an acute need of fracture surgery to assess whether the patient is benefited by specific SARS-CoV-2 treatment or further optimization perioperatively. Of the known factors that influence general patient outcomes with SARS-CoV-2 are treatment with dexamethasone and improvements in intensive care routines [16, 17]. How these developments pertain to trauma and orthopaedic patients with concomitant SARS-CoV-2, however, needs to be investigated further when best employed.

Sampling of nasopharynx and qRT-PCR rapidly became the standard for assessment of SARS-CoV-2 at Karolinska University Hospital as with many other hospitals but false negative results might have contributed to an underestimation of both infected patients and its influence on patient outcomes [18]. As this study could include qRT-PCR sample results also after patient discharge, due to the VAL database covering all testing in inpatient and outpatient care in specialist and primary care in Stockholm County, the percentage of false negative patients is likely lower. However, as 28–31% of infected patients remain asymptomatic [19], an underestimation of SARS-CoV-2 patients is inevitable despite a high testing rate in Stockholm County and should be taken into consideration when assessing its influence on study outcomes [19].

### Strengths and limitations

Drawing general conclusions from these findings may be hampered by the single-city study design. Although the study is population-based and the findings are consistent across the hospitals of Stockholm County, each with slightly differing perioperative protocols and indications for surgery, the demographic in Stockholm differs slightly to the other parts of Sweden, as is common for capital cities. The data coverage of the estimated 2.4 million inhabitants of Stockholm County with the VAL database is, however, extensive, with complete follow-up records of patients regarding SARS-CoV-2 status and mortality also after discharge from the main six emergency hospitals.

While SARS-CoV-2 cases in Stockholm have reached 273,337, SARS-CoV-2-positive fracture patients luckily are relatively few, thus far limiting extensive subgroup and pairwise analysis in several fracture types. Furthermore, the dataset did not allow for full resolution of orthopaedic procedures, which for combined orthopaedic procedure codes such as knee and lower leg precluded further statistical analysis of the two separate fracture regions.

## Conclusions

Perioperative SARS-CoV-2 infection increased 30-day mortality risk and hospital length of stay for acute fracture patients requiring inpatient surgical care. Pre- and postoperative infection were both associated with similar increases in mortality risk.

## Supplementary Information

Below is the link to the electronic supplementary material.

Supplementary file1 (DOCX 35 kb)

## Data Availability

No data are available. Data from deidentified the administrative health registry are not freely available due to protection of the personal integrity of the participants.

## Abbreviations

CI: Confidence interval
COVID-19: Coronavirus disease 2019
IQR: Inter-quartile range
OR: Odds ratio
qRT-PCR: Real-time quantitative reverse transcription polymerase chain reaction
SARS-CoV-2: Severe acute respiratory respiratory syndrome coronavirus 2
