# Supplementary material for: Influence of perioperative SARS-CoV-2 infection on mortality in orthopaedic inpatients with surgically treated traumatic fractures
Source: Eur J Orthop Surg Traumatol. 2022 Mar 24;33(4):1043–9. doi: 10.1007/s00590-022-03226-x (PMC8943350; doi:10.1007/s00590-022-03226-x)
Supplement: Supplementary file 1 — Supplementary file1 (DOCX 35 kb) [file 590_2022_3226_MOESM1_ESM.docx]

**Supplementary material for**

Influence of perioperative SARS-CoV-2 infection on mortality in orthopaedic inpatients with traumatic fracture procedures

**Authors:**

Mathias Granqvist, MD., Ph.D., Pontus Hedberg, MD., Pontus Naucler, MD., Ph.D., Anders Enocsson, MD., Ph.D.

**Correspondence to:** [Granqvist.mathias@gmail.com](mailto:Granqvist.mathias@gmail.com)

**Table of Contents**

| **Content** | **Title** | **Page** |
| --- | --- | --- |
| Table S1. | Orthopaedic procedure codes and ICD-10 codes for study cohort definition | 3 |
| Table S2. | Definition of co-morbidity categories according to ICD-10 codes and drug-dispensing | 4 |
| Figure S1. | Distribution of study cohort per calendar month of admission and proportion of healthcare episodes with perioperative SARS-CoV-2 infection | 5 |

**Table S1. Orthopaedic procedure codes and ICD-10 codes for study cohort definition**

| **Code type** | **Code** | **Description** |
| --- | --- | --- |
| Procedure code, KVÅ | NAJ | Spinal fracture surgery |
| Procedure code, KVÅ | NBB | Shoulder prosthesis surgery |
| Procedure code, KVÅ | NBJ | Shoulder or overarm fracture surgery |
| Procedure code, KVÅ | NBQ | Shoulder or overarm amputation |
| Procedure code, KVÅ | NCB | Elbow prosthesis surgery |
| Procedure code, KVÅ | NCJ | Elbow or underarm fracture surgery |
| Procedure code, KVÅ | NCQ | Underarm amputation |
| Procedure code, KVÅ | NDJ | Wrist or hand fracture surgery |
| Procedure code, KVÅ | NEJ | Pelvic fracture surgery |
| Procedure code, KVÅ | NFB | Hip prosthesis surgery |
| Procedure code, KVÅ | NFJ | Femur fracture surgery |
| Procedure code, KVÅ | NFQ | Femoral amputation |
| Procedure code, KVÅ | NGJ | Knee or lower leg fracture surgery |
| Procedure code, KVÅ | NGQ | Knee or lower leg amputation |
| Procedure code, KVÅ | NHJ | Foot or toe amputation |
| Diagnosis code, ICD-10 | S00-S09 | Injuries to the head |
| Diagnosis code, ICD-10 | S10-S19 | Injuries to the neck |
| Diagnosis code, ICD-10 | S20-S29 | Injuries to the thorax |
| Diagnosis code, ICD-10 | S30-S39 | Injuries to the abdomen, lower back, lumbar spine and pelvis |
| Diagnosis code, ICD-10 | S40-S49 | Injuries to the shoulder and upper arm |
| Diagnosis code, ICD-10 | S50-S59 | Injuries to the elbow and forearm |
| Diagnosis code, ICD-10 | S60-S69 | Injuries to the wrist and hand |
| Diagnosis code, ICD-10 | S70-S79 | Injuries to the hip and thigh |
| Diagnosis code, ICD-10 | S80-S89 | Injuries to the knee and lower leg |
| Diagnosis code, ICD-10 | S90-S99 | Injuries to the ankle and foot |
| Diagnosis code, ICD-10 | T00-T07 | Injuries involving multiple body regions |
| Diagnosis code, ICD-10 | T08-T14 | Injuries to unspecified part of trunk, limb or body region |

Patients were included in the study if they had any of the procedure codes and any of the diagnosis codes registered. For patients with only foot or ancle procedures performed, at least two procedures had to be performed.

**Table S2. Definition of co-morbidity categories according to ICD-10 codes and drug-dispensing**

| **Co-morbidity category** | **ICD-10 ^a^** | **ATC-codes (drug dispensing)** |
| --- | --- | --- |
| Cancer | C00-C26, C30-C34, C37-C39, C40-C96 |  |
| Cerebrovascular disease | I60-I69 |  |
| Chronic kidney disease | N18 |  |
| Diabetes mellitus (type 1 or 2) | E10-E14 |  |
| Heart disease | I05-I08, I20-I22, I24-I28, I34-I37, I42, I44-I50 |  |
| Immunosuppression | B20-B24, C00-C26, C30-C34, C37-C39, C40-C96, D70-D72, D73.0, D80-D84, N18 | OR if a person had two or more prescriptions the last year AND if a person had one or more prescriptions the last three months (L04, H02) |
| Liver disease | K70.0-K70.4, K70.9, K71-K73, K74.0-K74.6, K75-K77 |  |
| Lung disease | J43, J44.1-J44.9, J45-J47, J60-J70, J80-J84, J92-J96, J98-J99 | OR if a person had two or more prescriptions the last year (R03) |
| Neurological disease | G10-G14, G20-G26, G30-G32, G35-G73, G80-G83, G90-G99 |  |
| Obesity | E65-E68 |  |
| Substance use | F10-F19 |  |

a. The assessment window was from five years to one day before hospital admission.

**Figure S1. Distribution of study cohort per calendar month of admission and proportion of healthcare episodes with perioperative SARS-CoV-2 infection**
